# A genome-wide *in situ *hybridization map of RNA-binding proteins reveals anatomically restricted expression in the developing mouse brain

**DOI:** 10.1186/1471-213X-5-14

**Published:** 2005-07-20

**Authors:** Adrienne E McKee, Emmanuel Minet, Charlene Stern, Shervin Riahi, Charles D Stiles, Pamela A Silver

**Affiliations:** 1 Department of Systems Biology, Harvard Medical School, Boston, MA 02115 USA; 2 Department of Cancer Biology, The Dana-Farber Cancer Institute, Boston, MA 02115 USA; 3 URBC-FUNDP, 61 rue de Bruxelles, 5000 Namur, Belgium; 4 Department of Microbiology and Molecular Genetics, Harvard Medical School, Boston, MA 02115 USA

## Abstract

**Background:**

In eukaryotic cells, RNA-binding proteins (RBPs) contribute to gene expression by regulating the form, abundance, and stability of both coding and non-coding RNA. In the vertebrate brain, RBPs account for many distinctive features of RNA processing such as activity-dependent transcript localization and localized protein synthesis. Several RBPs with activities that are important for the proper function of adult brain have been identified, but how many RBPs exist and where these genes are expressed in the developing brain is uncharacterized.

**Results:**

Here we describe a comprehensive catalogue of the unique RBPs encoded in the mouse genome and provide an online database of RBP expression in developing brain. We identified 380 putative RBPs in the mouse genome. Using *in situ *hybridization, we visualized the expression of 323 of these RBP genes in the brains of developing mice at embryonic day 13.5, when critical fate choice decisions are made and at P0, when major structural components of the adult brain are apparent. We demonstrate i) that 16 of the 323 RBPs examined show neural-specific expression at the stages we examined, and ii) that a far larger subset (221) shows regionally restricted expression in the brain. Of the regionally restricted RBPs, we describe one group that is preferentially expressed in the E13.5 ventricular areas and a second group that shows spatially restricted expression in post-mitotic regions of the embryonic brain. Additionally, we find a subset of RBPs that share the same complex pattern of expression, in proliferating regions of the embryonic and postnatal NS and peripheral tissues.

**Conclusion:**

Our data show that, in contrast to their proposed ubiquitous involvement in gene regulation, most RBPs are not uniformly expressed. Here we demonstrate the region-specific expression of RBPs in proliferating vs. post-mitotic brain regions as well as cell-type-specific RBP expression. We identify uncharacterized RBPs that exhibit neural-specific expression as well as novel RBPs that show expression in non-neural tissues. The data presented here and in an online database provide a visual filter for the functional analysis of individual RBPs.

## Background

The ordered production and differentiation of cell types that occurs during nervous system (NS) development relies upon tightly regulated gene expression. In neural cells, spatial and temporal gene regulation occurs through both transcriptional and post-transcriptional mechanisms. While the transcriptional networks that direct neural cell fate and govern cell shape, position, and connectivity have been well studied [1-3], the post-transcriptional influences on neural development and gene expression are less well understood.

At the core of post-transcriptional gene regulation are RNA-binding proteins (RBPs). Proteins containing canonical RNA-binding domains (RBDs) are involved in numerous steps of nuclear and cytoplasmic RNA processing [4]. Through mRNA capping, splicing, editing, polyadenylation and nonsense-mediated decay, RBPs modulate the diversity of transcribed genes [4-6]. RBPs also affect the processing of non-coding RNAs [7]. Specific RBPs additionally enable asymmetric RNA distribution and translational regulation [8-10], two phenomena that are critical for affecting localized protein synthesis [11,12].

The importance of post-transcriptional processing in NS gene regulation is underscored by functional examples of specific RBPs [13,14]. For instance, the neuronal-specific factor Nova-1 regulates splicing of pre-mRNAs that encode components of inhibitory synapses [15]. Mice lacking Nova-1 die postnatally due to aberrant regulation of apoptotic neuronal death [16]. As a second example, RBPs encoded by the quaking and Musashi loci promote glial cell fate [17] and CNS stem cell self-renewal [18] by stabilizing transcripts involved in cell differentiation. Thirdly, the fragile X mental retardation protein, members of the ELAV/Hu protein family, and the Staufen proteins are involved in targeting and translational regulation of dendritic transcripts [19-21]. Additionally, the finding that long-term memory requires *de novo *protein synthesis highlights the significance of post-transcriptional processes in neural function [22,23].

Despite our knowledge of several key RBPs, much of the understanding of RBPs in the brain comes from studies of adult animals or neural cell lines. Thus, how the functional class of RBPs contributes to the positioning, growth, and diversification of cells in the developing brain is not well understood. One step towards increasing our understanding RBPs is to resolve where they are expressed. Here, we utilize the approach of *in situ *hybridization mapping [24-26] to investigate the expression of 323 RBPs within the developing mouse brain. Two stages of development were characterized, embryonic day 13.5 (E13.5), when critical cellular fate choice decisions are made and postnatal day 0 (P0), when the major structural components of the brain are apparent. We find that, in contrast to their proposed ubiquitous involvement in gene regulation, most RBPs are not uniformly expressed. The majority of RBPs profiled demonstrates spatially restricted expression in the brain or in other peripheral tissues examined. The data presented here and in an online database afford a visual filter for the functional analysis of individual RBPs in the developing mammalian NS.

## Results

### Mouse RBPs were identified according to gene sequence

The RNA recognition motif (RRM), the hnRNP K-homology (KH) domain, and the double-stranded RNA-binding domain (dsRM) are evolutionarily conserved, well-characterized domains known to bind either single or double-stranded RNA [27-29]. Sequence similarity searches and structural analyses of these domains have led to the ability to predict other RBPs based on primary coding sequence [29]. To identify unique genomic loci that encode putative RBPs in the mouse genome, we analyzed existing public [30,31] and private [32] databases for sequences containing one or more RBD. Candidates were classified as RBPs only if their predicted protein sequence contained a Protein Families Database (Pfam)-defined RBD [31].

We identified 290 genes harboring one or more RRM, KH, or dsRM sequences. We also identified 32 genes encoding other domains shown to interact with RNA, including the zinc knuckle, G-patch, PIWI, DEAD box RNA helicase, and TUDOR domains. Finally, as the absence of a canonical RBD does not preclude interaction with RNA, we sought 58 additional genes known or predicted to be associated with RNA processing. In total, this collection contains 380 putative RBPs. Additional file 1 lists the number of genes, per RBD, identified and analyzed by *in situ *hybridization. A list of all genes and primer sequences is given in Additional file 2.

### RBP expression in the developing mouse brain was analyzed by *in situ *hybridization

To localize RBP expression, we preformed *in situ *hybridization on whole head tissue sections of E13.5 embryos and P0 mice. We designed gene-specific primers to produce 400–700 bp probes for 340 candidate RBPs. These primer sets were used to perform PCR on cDNA prepared from embryonic or P0 mouse brains. A small number of probes were obtained from mouse intestine, liver, kidney, or testes cDNA. 323 genes (95%) showed positive PCR products (data not shown). Following subcloning, anti-sense digoxygenin-labeled riboprobes were prepared and hybridized against coronal head and transverse upper-body sections (to include the brain and spinal cord, respectively). Digital images of the entire *in situ *hybridization set have been deposited in the Mahoney RNA-Binding Protein Expression Database [33].

### RBPs exhibit restricted expression in the developing mouse brain

Several neural-specific RBPs have been identified, yet how many others demonstrate this degree of specificity is unknown. Of the genes examined we found 16 RBPs (listed in Additional file 2) that exhibit NS-restricted expression in the tissues analyzed. Among this list are known examples of neuronal-specific RBPs including Nova-1 [34], the ELAV/Hu proteins B, C, and D [35], and Ataxin 2 binding protein 1 (A2bp1) [36] but additionally include putative RBPs for which expression has not been reported. With the exception of one gene that was only detected at E13.5, all (15/16) of these RBPs appear brain or NS-specific at both developmental stages in the tissues analyzed. Overall, these RBP encoding genes are not limited in expression to one brain region but are found in multiple brain or NS structures.

### RBPs show spatially restricted expression in anatomically distinct brain regions

We find that greater than half of the RBPs profiled exhibit spatially restricted expression. Of the 323 genes examined, 221 demonstrate localized, enriched expression in one or more discrete brain regions in addition to detectable expression in non-NS tissues. We divided the E13.5 and P0 CNS into five and eight general areas for annotation, respectively: the E13.5 precortical area, the striatum (and other basal ganglia), the periventrical areas, hindbrain, and spinal cord, as well as the P0 cortex, striatum, hippocampus, thalamus, hypothalamus, midbrain, hindbrain, and spinal cord. The presence or absence of expression for each RBP was analyzed visually at each location and is annotated in Additional file 3. Very few of the 221 RBPs with spatially restricted expression patterns were expressed in only one brain region, however most (73%) showed restricted expression at both developmental stages (Additional file 3).

**Figure 1:**
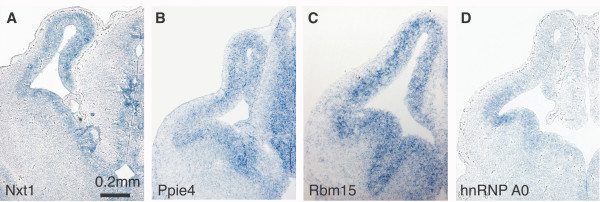
**RBP expression in proliferative zones of the E13.5 mouse forebrain. ***In situ *hybridization patterns for four RBPs on sections through the forebrain of E13.5 mice. Labels indicate Locuslink gene names. All images show the same magnification.

We observe multiple RBPs that demonstrate region-specific expression in the E13.5 ventricular areas. Shown in Figure 1 are representative RBP genes that are transcribed in mitotically-active cells in the neuroepithelia of the developing telencephalon. Among the RBPs expressed in this region occupied by neural progenitor cells, we find examples of mRNA export factors in addition to putative splicing factors and transcriptional regulators (Fig. 1). In all instances, expression in the embryonic lateral ventricular zone is accompanied by expression in the periventricular areas of the 3^rd ^and 4^th ^E13.5 ventricles and often by heightened expression in the P0 subventricular zone [33]. Notably, we observed this pattern of expression for the dsRM-containing Musashi proteins [33]. Our results are consistent with the documented expression of Msi1 and Msi2 [37,38].

**Figure 2:**
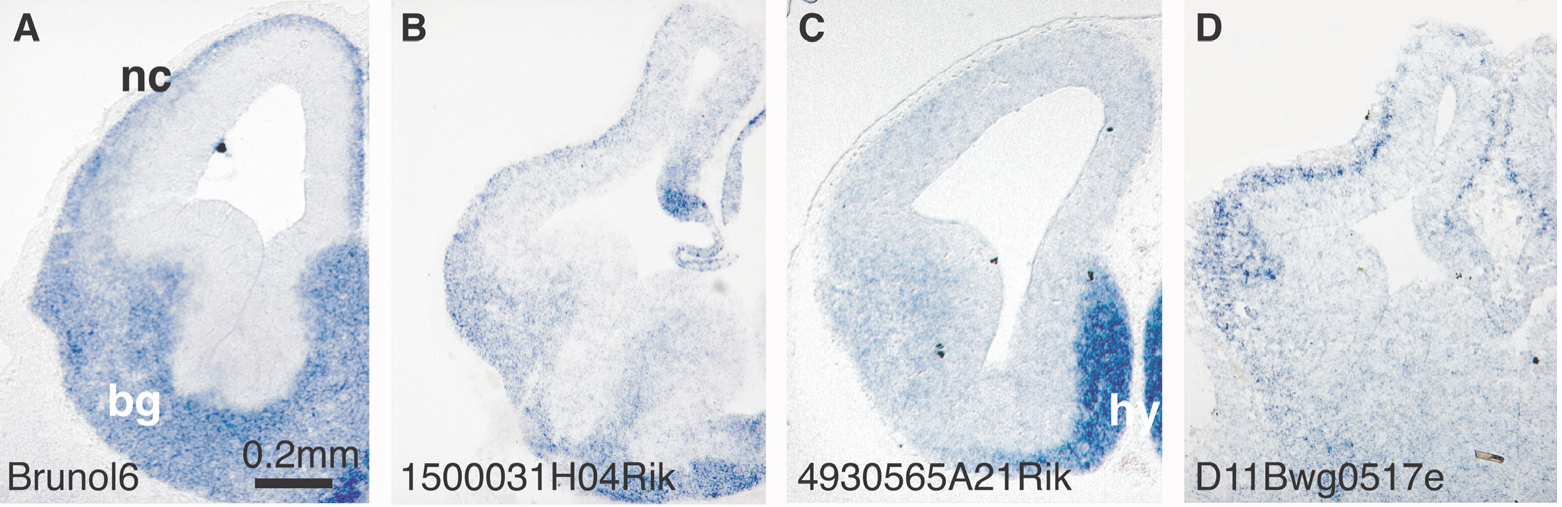
**RBP expression in post-mitotic areas of the E13.5 mouse forebrain. ***In situ *hybridization patterns for four RBPs on sections through the forebrain of E13.5 mice. Labels indicate Locuslink gene names. bg, basal ganglia; hy, hypothalamus; nc, neocortex. All images show the same magnification.

Multiple RBPs show restricted expression in post-mitotic regions of embryonic brain. Presented in Figure 2 are examples of four putative RBPs that demonstrate region-specific expression in areas containing post-mitotic neurons. Transcripts of the genes encoding the RRM protein Brunol6 and the predicted zinc-knuckle protein 1500031H04Rik appear pan-neuronal at both developmental stages (Fig. 2A, 2B and [33]). Expression of the RRM-containing RIKEN gene 4930565A21 is most pronounced in the ventral telencephalon, while D11Bwg0517e is found in the precortical layer, the thalamic area and hindbrain (Fig. 2C, 2D and [33]). Among the genes that occupy post-mitotic regions of the developing brain we additionally observe members of the ELAV/Hu family as well as other RBPs that have well-documented neuronal expression [34,35].

### RBPs demonstrate cell-type specific expression in the P0 mouse retina

**Figure 3:**
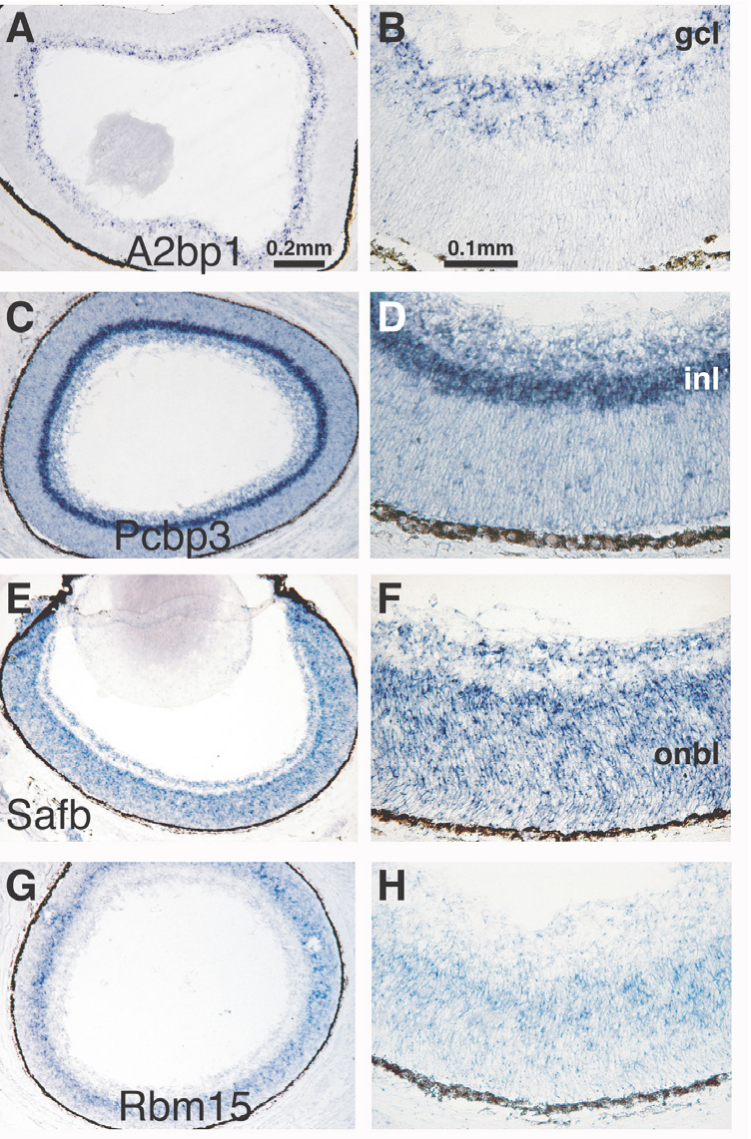
**Diversity of RBP expression in major cellular subtypes of the P0 retina. ***In situ *hybridization for four representative RBPs that exhibit laminar-specific expression in the P0 mouse retina. Labels indicate Locuslink gene names. A, B) A2bp1, C, D) Pcbp3, E, F) Safb, G, H) Rbm15. Panels A, C, E, and G show the same magnification. Panels B, D, F, and H show the same magnification. gcl, granule cell layer; inl, inner nuclear layer, onbl; outer neuroblastic layer.

As our *in situ *hybridization analyses were performed on sections through whole head, we were able to visualize RBP expression in the developing retina. The vertebrate retina provides a distinctive system for studying CNS development as its seven major neural cell types are readily distinguished from one another by their morphology and laminar position [39]. Shown in Figure 3 are examples of the diversity of RBP expression in the P0 retina. The RRM-containing A2bp1 is expressed in the retinal ganglion cell layer (GCL), which contains primarily retinal ganglion cells and a small number of displaced amacrine cells (Fig 3A, 3B). The KH-domain encoding gene poly(rC) binding protein 3 (Pcbp3) shows dramatically enriched expression in the inner nuclear layer (INL) (Fig. 3C and 3D), possibly indicating localization to the bipolar neuron cell bodies that occupy the scleral portion of the INL. Notably, both A2bp1 and Pcbp3 show restricted expression in post-mitotic regions of the E13.5 and P0 brain [24,36]. Transcripts of the RRM-encoding scaffold attachment factor B (Safb) and of the three-RRM containing SPOC gene Rbm15 are expressed in the outer neuroblastic layer of the retina (Fig. 3E–H). Safb, but not Rbm15, is additionally expressed in the GCL, possibly in the Müller glia. Both Safb and Rbm15 show enriched expression in neuroepithelia of the ventricular zone (Fig. 1 and [33]).

### A systems-based view of RBP expression

**Figure 4:**
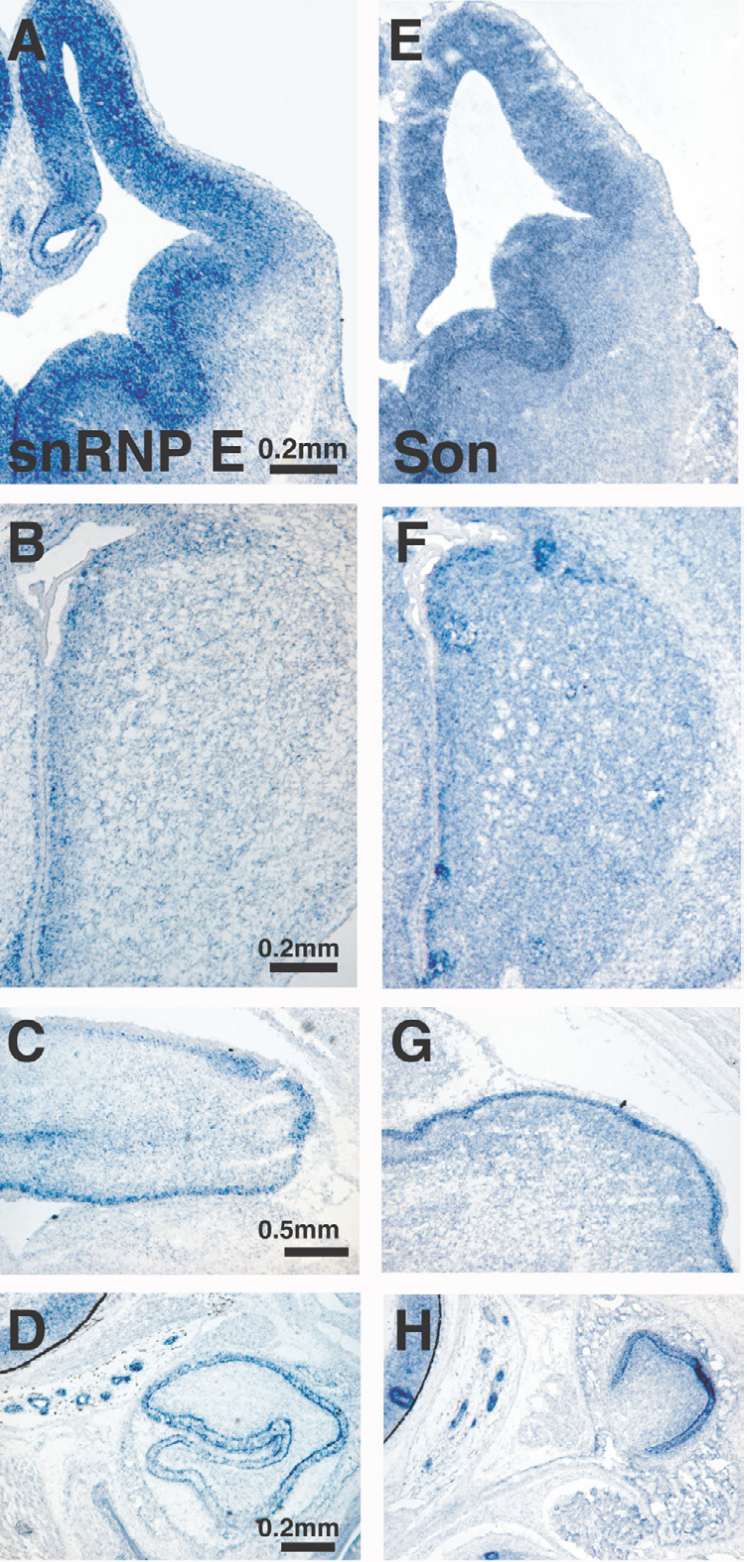
**Representative examples of RBP synexpression in E13.5 and P0 mouse tissues. **snRNP E and Son are transcribed in the perventricular areas of the E13.5 brain (A, E), in the P0 subventricular area of the lateral ventricle (B, F), in the external granule layer of the P0 cerebellum (C, G), as well as in postnatal developing teeth (D, H).

Gene regulation by RBPs is believed to occur through coordinated, combinatorial interactions with RNA. During the course of this study we identified multiple RBPs that are coordinately expressed in the brain and other tissues. We find 48 genes (listed in Additional file 4) that show elevated expression in proliferating areas of the embryonic and postnatal brain as well as in postnatal nasal epithelia, teeth, and thymus. Presented in Figure 4 are expression data for snRNP E and Son, two representative examples of this "synexpression group" of genes that share a similar, complex pattern of expression. Further examples are shown in Additional file 5. This same expression distribution has been observed for the polypyrimidine tract-binding protein, PTBP1, and our data are consistent with previous findings [40]. Notably, the protein products of many of the genes listed are understood to interact either physically or genetically.

### RBPs show restricted expression in non-NS tissues

**Figure 5:**
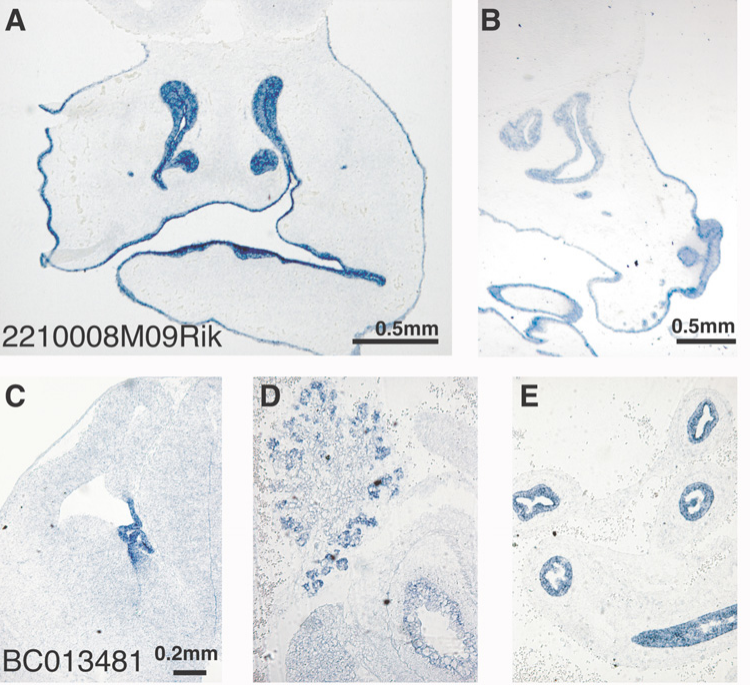
***In situ *hybridization profiling uncovers the non-neural, restricted expression of novel RBPs. **Data from ISH performed on (A, C) coronal E13.5 and on (B, D, E) E15 sagittal sections are presented for RRM-encoding RBPS. A, B) The Riken gene 2210008M09 is transcribed in epithelia covering the facial skeleton. C-E) BC013481 is detected in the choroid plexus, in the intestinal lining, and in the lining of the placenta. Panels C-E show the same magnification.

As our analyses were performed on whole head and upper thoracic tissues, our data provide detailed information about RBP expression in developing cranial facial tissues. We identified putative RBPs that display tissue-restricted expression in non-NS structures (listed in Additional file 3). Figure 5 presents *in situ *hybridization results for two RRM-encoding transcripts that show highly restricted expression in different epithelial tissues. The Riken gene 2210008M09 is transcribed in epithelia covering the facial skeleton (Fig. 5A, 5B), while the gene BC013481 is expressed in the choroid plexus (Fig. 5C) and in the lining of the intestine and placenta (Fig. 5D, 5E).

## Discussion

Neural cells utilize multiple forms of post-transcriptional gene regulation. While RBPs are believed to be potent modulators of post-transcriptional processes, little is known about how this functional class is expressed in the developing brain. As a first step towards increasing our knowledge of RBPs we chose to investigate the spatial and temporal expression of genes that encode motifs known to interact with RNA. We find a small set of RBPs that show neural-specific expression in the tissues analyzed. An even greater number of RBP genes however demonstrate spatially restricted expression in distinct regions of the developing brain.

Within the CNS, most of the RBPs examined show non-uniform, heightened expression in anatomically discrete structures. Tissue differences in the expression levels of individual genes could indicate distinctive protein requirements among cell types, beyond that of tissue-specific RBPs [41]. There is precedent for differential requirements of individual RBPs, as tissue-specific RNA splicing is achieved partly through combinatorial, stoichiometric differences among splicing factors within various cells [42]. It is from this local enrichment within different cell types or tissues that we can begin to hypothesize as to the functional significance of individual genes as well as to the importance of groups of similarly expressed RBPs.

Our study has identified RBPs that display spatially restricted expression in distinct regions of the developing mouse brain. One set of RBPs (Fig. 1) is found in the E13.5 ventricular areas. A second set demonstrates spatially restricted expression in post-mitotic regions of E13.5 brain (Fig. 2). Based on their pattern of expression, these RBPs may have roles in neural proliferation, cell fate choice and cell migration, or in neuronal function, respectively. We also identified novel RBPs that are expressed in tissues of mesodermal and endodermal origin (Fig. 5). The highly restricted expression of these genes may indicate an explicit role for these RBPs in their respective epithelia. Additionally, the cell-type specificity RBPs found in the P0 retina (Fig. 3) illustrates the diversity of RBP expression. The specialized expression of these RBPs may be indicative of a dedicated function in the specified tissues.

By visual inspection of *in situ *hybridization data, we find a subset of RBPs that are coordinately expressed in multiple tissue types. These genes display heightened expression in the periventricular areas of the E13.5 brain and spinal cord as well as marked expression in the external granule layer of the P0 cerebellum, the lateral subventricular zones, and in teeth, nasal epithelia, and thymus (Fig. 4, Additional file 5, [33]). While not excluded from post-mitotic tissues, these RBPs are predominately expressed in structures that are undergoing cell division.

Notably, the term 'synexpression group' has been used to describe collections of genes that function in a common process and share a similar complex spatial expression pattern in multiple tissues [43]. Among the synexpression group identified here we find examples of RBPs that are known to interact either physically or genetically (Additional file 4). For example, PTBP1 binds the splicing factors PSF [44] and hnRNP L [45] while SF2/ASF and hnRNP A1 select for 5' exon or exclusion or inclusion, respectively [46]. Our data provide visual support to a growing body of evidence that functionally-related transcripts are post-transcriptionally co-regulated [47].

Although the significance of certain splicing and mRNA export factor enrichment in proliferating regions is not known, data from multiple studies point to a role for RBPs in cell proliferation. During hippocampal development expression levels of RBPs were found to be high and then to dramatically decrease, as neurons transition from a proliferating to a post-mitotic state [48]. A number of RBPs were also identified as highly expressed in a molecular characterization of gastric epithelial progenitor cells [49,50]. Furthermore, protein levels of hnRNPs and snRNPs were found to be down-regulated upon stimulated growth inhibition of myeloid cells [51]. Therefore, it is likely that a role for RBPs during cell proliferation and cell fate determination exists in multiple tissue types.

## Conclusion

In summary, the data presented here provide new insight into how a distinct functional gene class is expressed in the developing NS. We find that RBPs demonstrate region-specific as well as cell-type specific expression. In addition, we find that specific, proliferating regions of the embryonic and postnatal NS and peripheral tissues are similar in the expression of certain RBPs. These data serve as a starting point for functional investigations into the roles of RBPs in neural development and physiology.

## Methods

### *In silico *RBP identification

Putative RBP gene sequences were identified by homology-based whole genome screening using public and private databases: Celera Panther Families, Protein Families Database (Pfam), and Genbank [30-32]. Classification as an RBP was based on the presence of one or more RRM, KH, or dsRMs, as defined by Pfam databases [31]. Databases were also mined for zinc-knuckle, G-patch, PIWI, DEAD-box helicase and Tudor domain-containing sequences and for known factors involved in mRNA splicing, editing, transport, and stability. Genes with multiple RNA-binding domains were assigned to a single subfamily. Unique gene identity was verified by LocusID numbers. As of March 1, 2004, a total of 357 unique genes were identified from these sources. An additional 26 RRM, KH, and dsRM proteins have been identified as of March 7, 2005.

### PCR primer design

PCR primer pairs were designed for each identified RNA-binding protein locus. PCR primer sequences were designed with approximately 60% GC content, spanning 400–700 base pairs of primarily the gene's coding sequence. Additional primer pairs were designed for targets that did not initially yield PCR products.

### Cloning

Total RNA was obtained from E13.5, P0, or adult C57/BL6 mouse brains (Charles River Laboratories) by Trizol extraction (Invitrogen). Reverse transcription was performed using Superscript II reverse transcriptase and oligo-dT (Invitrogen). PCR was performed with cDNA templates using 40 cycles, 60–65°C annealing temperature, and Platinum Taq (Invitrogen) as polymerase. For a few genes, PCR was performed with cDNA templates prepared from adult brain, kidney, gut, liver, or testis tissues. Positive PCR products were cloned into TA cloning vectors (Invitrogen) and verified by restriction digest or DNA sequencing.

### Probe synthesis

Gene fragments from verified plasmids were amplified by PCR using plasmid specific primers. Digoxigenin-labeled RNA probes were made, using PCR products as template and T7 or SP6 RNA polymerases (Roche). cRNA probes were ethanol precipitated and quantified by spectrophotometry.

### Tissue preparation

E13.5 embryos were directly fixed overnight in 4% paraformaldehyde (0.1M PBS). P0 mice were transcardially perfused with 4% paraformaldehyde (0.1M PBS) and postfixed overnight at 4°C. After fixation, embryos and P0 mice were transferred to 20% sucrose overnight. The head, neck, and trunk were embedded separately in OCT (Tissue-Tek) on dry ice and stored at -80°C. Serial cryostat sections (14 μm) were cut and mounted on Superfrost Plus slides (Fisher). Ten and twenty adjacent sets of sections were prepared from E13.5 embryos and P0 mice, respectively, and were stored at -20°C until use.

### Section *in situ *hybridization

*In situ *hybridization was performed according to Gray et al. [25]. Following pretreatment (Proteinase K), slides were pre-hybridized for 1h at 65°C in hybridization solution (50% formamide (Ambion), 5X SSC, 0.3 mg/ml yeast tRNA (Sigma), 100 μg/ml heparin (Sigma), 1X Denhardt's (Sigma), 0.1% tween, 5 mM EDTA). P0 and E13.5 brain sections were hybridized overnight with labeled RNA probe(0.8–1.2 μg/ml) at 65°C, washed in 2X SSC at 67°C, incubated with RNase A (1 μg/ml, 2X SSC) at 37°C, washed in 0.2X SSC at 65°C, blocked in PBS with 10% lamb sera, and incubated in alkaline phosphatase labeled anti-DIG antibody (Roche) (1:2000, 10% sera) overnight. Sections were washed and color was visualized using NBT and BCIP in alkaline phosphatase buffer (100 mM Tris pH 9.5, 50 mM MgCl2, 100 mM NaCl, 0.1% tween-20) containing 75 μg/ml NBT (BioRad), 600 μg/ml BCIP (Roche). Staining was stopped after visual inspection. Sections were washed, fixed in 4% paraformaldehyde, and coverslipped in glycerol [25].

### Image acquisition and RBP expression database

Images were acquired and analyzed as described [25]. Images were either scanned using a Nikon Coolscan 8000 slide scanner (4000 DPI) or digitally acquired using a Leica digital camera. Image levels have been modified in Photoshop (Adobe) for clarity. Full resolution scanned images were compressed using JPEG compression, quality 10, and have been deposited in the Mahoney RNA-Binding Protein Expression Database [33].

## Authors' contributions

AEM prepared tissue samples, performed data analysis and drafted the manuscript. EM performed data analysis and both EM and SR generated reagents, tissue samples, digitized the raw data, and helped build the website. CS contributed to the design of the study and prepared tissue samples. CDS and PAS conceived of the study, participated in its design and coordination and helped prepare the manuscript. All authors read and approved of the manuscript.

## Supplementary Material

Additional File 1 **RNA-binding proteins identified *in silico *and profiled by *in situ *hybridization. **List of annotated RNA-binding domains and the number of family members that were identified *in silico *and analyzed by *in situ *hybridization. Click here for file

Additional File 2 **List of 380 genes identified as putative RBPs in the mouse genome and analyzed in this study. **Columns indicate LocusID, gene name, type of RBD, primer sequences used to isolate the target cDNA, the size of the cDNA fragment, the presence call by PCR from E13.5 and P0 brain cDNA, cloning status ('c' indicates cloned, 'u' indicates uncloned, 'small' indicates that the target gene had less than 400 bp of unique sequence, 'na' indicates that cloning was not attempted), the RNA polymerase used to generate the anti-sense riboprobe, the tissue from which the cDNA was isolated (if not from E13.5 or P0 mouse brain), and whether the gene was analyzed by *in situ *hybridization ('x' indicates yes). Click here for file

Additional File 3 **Complete list of gene expression patterns for all *in situ *hybridizations performed. **Of the 323 RBPs examined, 221 showed restricted expression patterns in the brain. The remaining genes either show restricted expression in non-neural tissues, ubiquitous expression that is difficult to distinguish from background, or no expression. Caution is needed in interpreting the results. First, non-expression could be due to the sensitivity limit of non-radioactive *in situ *hybridization. Second, the background level of individual probes may differ. Third, some probes with high background hybridization may mask the real expression of the transcript. Fourth, we cannot rule out the possibility that some probes may show variable levels of background hybridization in different brain areas, resulting in a false positive signal. Columns **A-D **describe the LocusID, gene name, type of RBD, and number (internal Mahoney reference number). Columns **E and, L **(E13.5, P0 "**Informativity**"): "1" for restricted expression in the nervous system and "0" for either ubiquitous expression that is difficult to distinguish from background or no expression. As noted in Gray et al [25], some of the genes in the "0" category show uneven signals in different brain regions and are also annotated in the subsequent columns. Columns **F and M **(E13.5, P0 "**Specificity**"): "1" for restricted expression in neural tissues only, "2" for restricted expression in neural tissue with distinguishable expression in non-neural tissue, "3" for ubiquitous or no expression, and "4" for expression in non-neural tissues only. Columns **G-K and N-U **(E13.5, P0 "**Expression**"): "2" for expression, "1" for ubiquitous expression or background, "0" for no expression. Click here for file

Additional File 4 **RNA-binding proteins belonging to a synexpression group. **Complete list of RBPs that demonstrate a similar complex pattern of expression. Columns **A-D **describe the LocusID, gene name, type of RBD, and number (internal Mahoney reference number). Click here for file

Additional File 5 **Examples of RBP synexpression in E13.5 and P0 mouse tissues. **Additional examples of RBPs that share a similar pattern of expression. Shown are *in situ *hybridization results of expression in the periventricular areas of the E13.5 brain (A, E, I, M, Q), in the subventricular area of the P0 lateral ventricle (B, F, J, N, R), in the external granule layer of the P0 cerebellum (C, G, K, O, S), as well as in postnatal developing teeth (D, H, L P, T). A-D) Refbp1, E-H) hnRNP A1, I-L) PTBP1, M-P) Sfpq, Q-R) Hnrpl. Panels A, B, E, F, I, J, M, N, Q, R show the same magnification. Panels C, D, G, H, K, L, O, P, S, T show the same magnification. Click here for file
