# Supplementary figures and images for: A genome-wide in situ hybridization map of RNA-binding proteins reveals anatomically restricted expression in the developing mouse brain
Source: BMC Dev Biol. 2005 Jul 20;5:14. doi: 10.1186/1471-213X-5-14 (PMC1199591; doi:10.1186/1471-213X-5-14)

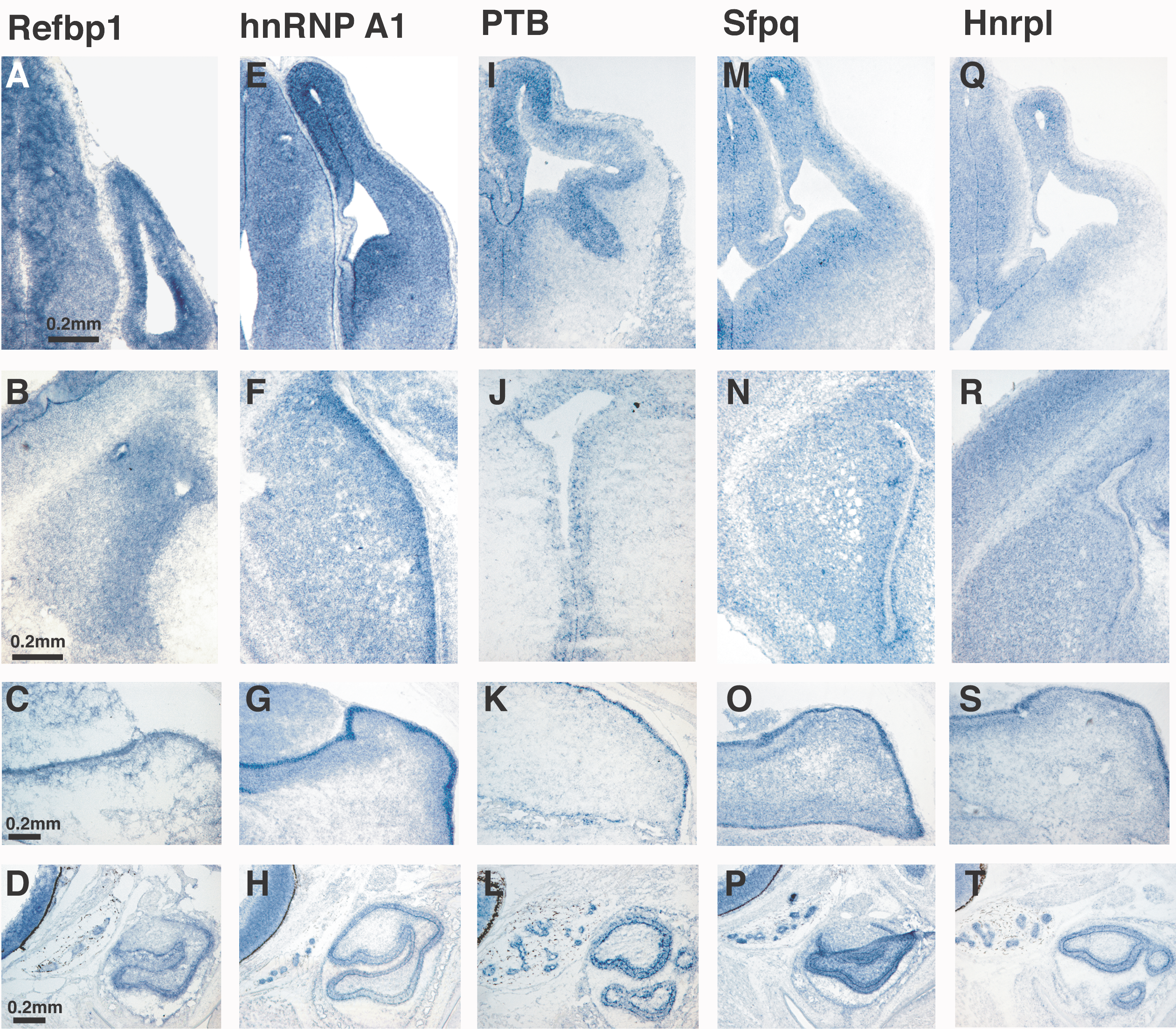

Supplement: Additional File 5 — Examples of RBP synexpression in E13.5 and P0 mouse tissues. Additional examples of RBPs that share a similar pattern of expression. Shown are in situ hybridization results of expression in the periventricular areas of the E13.5 brain (A, E, I, M, Q), in the subventricular area of the P0 lateral ventricle (B, F, J, N, R), in the external granule layer of the P0 cerebellum (C, G, K, O, S), as well as in postnatal developing teeth (D, H, L P, T). A-D) Refbp1, E-H) hnRNP A1, I-L) PTBP1, M-P) Sfpq, Q-R) Hnrpl. Panels A, B, E, F, I, J, M, N, Q, R show the same magnification. Panels C, D, G, H, K, L, O, P, S, T show the same magnification. [file 1471-213X-5-14-S5.png]
